# Multimodal data integration with machine learning for predicting PARP inhibitor efficacy and prognosis in ovarian cancer

**DOI:** 10.3389/fonc.2025.1571193

**Published:** 2025-06-04

**Authors:** Xi’an Xiong, Li Cai, Zhen Yang, Zhongping Cao, Nayiyuan Wu, Qianxi Ni

**Affiliations:** ^1^ The Affiliated Cancer Hospital of Xiangya School of Medicine, Central South University/Hunan Cancer Hospital, Changsha, China; ^2^ College of Pharmacy, Dali University, Dali, China; ^3^ Oncology Department of Xiangya Hospital, Central South University, Changsha, China; ^4^ Department of Oncology of the Second XiangYa Hospital, Central of South University, Changsha, China; ^5^ Hunan Key Laboratory Of The Research and Development of Novel Pharmaceutical Preparations, Changsha Medical University, Changsha, China

**Keywords:** PARP inhibitors (PARPi), prognostic factor, ovarian cancer, machine learning, prediction model

## Abstract

**Background:**

Poly(ADP)-ribose polymerase inhibitors (PARPi) have brought a significant breakthrough in the maintenance treatment of ovarian cancer. However, beyond BRCA mutation/HRD, the direct impact of other prognostic factors on PARPi response and prognosis remains inadequately characterized.

**Methods:**

We assessed PARPi prognostic factors from clinical characteristics, pathological findings, and biochemical indicators from 251 ovarian cancer patients. Cox univariate and multivariate analyses were employed to identify the factors which influencing PARPi efficacy and patients prognosis. Feature screening was conducted using correlation analysis, significance analysis, Variance Inflation Factor (VIF), and Elastic Net stability analysis. Patient-specific efficacy and prognosis prediction models were then constructed using various machine learning algorithms.

**Results:**

Total bile acids (TBAs) and CA-199 present as an independent risk factor in Cox multivariate analysis for primary and recurrent ovarian cancer patients respectively (P < 0.05). TBAs emerged as a risk factor, with each unit increase associated with a 10% rise in recurrence risk. The best-performing model has an AUC of 0.79 ± 0.09 and an AUC of 0.72 ± 0.03 for primary and recurrent ovarian cancer patients respectively. External validation(n=36) in multicenter cohorts maintained robust performance with AUC of 0.74 and an AUC of 0.70 for primary and recurrent ovarian cancer patients respectively.

**Conclusions:**

We identified TBAs and CA-199 as a significant prognostic factor in primary and recurrent ovarian cancer patients respectively. The integration of multimodal data with machine learning holds significant potential for enhancing prognosis prediction in PARPi treatment for ovarian cancer.

## 1 Background

Ovarian cancer, often referred to as the “silent killer”, presents the highest mortality rate among gynecological malignancies (1). The standard treatment for ovarian cancer includes cytoreductive surgery followed by systemic platinum-taxane combination chemotherapy (2). Although most patients achieve clinical remission with initial therapy, about 70% of patients may relapse within 2 to 3 years and eventually develop platinum resistance. The 5-year survival rate remains approximately 40% (3–5). The introduction of PARPi has significantly advanced the treatment of ovarian cancer. PARPi can induce apoptosis and death in cancer cells with BRCA mutations or other homologous recombination deficiencies (HRD) through a mechanism known as the “synthetic lethal” effect (6–8). Numerous studies have demonstrated that PARPi can significantly improve progression-free survival (PFS) in patients with BRCA-mutated ovarian cancer (9–12). Consequently, BRCA gene mutations or HRD are commonly utilized as biomarkers for the application of PARPi therapy (13). However, clinical evidence indicates that patients without BRCA mutations or HRD can also derive benefits from PARPi therapy (14–16). And more than 40% patients with BRCA mutations or HRD failed to benefit from PARPi (17, 18). One potential reason for this discrepancy is the insufficient consideration of various prognostic factors in clinical studies evaluating PARPi efficacy in ovarian cancer. These studies often fail to thoroughly investigate which clinicopathological factors might serve as reliable prognostic indicators for PARPi response. Beyond BRCA mutations and HRD, few prognostic factors are currently utilized to guide the clinical application of PARPi. A meta-analysis by Huang et al., encompassing 20 prospective studies, identified BRCA mutation, HRD-positive status, and platinum sensitivity as significant prognostic factors for PARPi efficacy in ovarian cancer; however, other clinicopathological variables did not show a significant predictive value for PFS (19). In contrast, Yusuke et al. demonstrated that HRD status, age, pathological stage, and residual disease status post-cytoreductive surgery are critical prognostic factors for ovarian cancer (20). Bile acids (BAs) have been recognized for their potential role in preventing ovarian cancer by inhibiting proliferation, invasion, and epithelial-mesenchymal transition, as well as enhancing chemotherapy efficacy (21–23). Furthermore, BAs can modulate the expression and activity of multiple PARP enzymes, which may ultimately improve patient survival (24, 25). Lamkin et al. found that higher glucose levels were associated with shorter survival time in ovarian cancer patients in univariate analysis (HR = 1.88; P < 0.05). Multivariate analysis, adjusted for tumor stage, showed that higher glucose levels were associated with shorter survival time (HR = 2.01; P=0.04) and disease-free interval(HR = 2.32; P < 0.05) (26). Additionally, Zhu et al. identified elevated postoperative CA-199 as an independent risk factor for both PFS and overall survival (OS) in patients with normal postoperative CA-125 levels. The combination of postoperative CA-199 and CA-125 levels offers significant prognostic value for patients with ovarian clear cell carcinoma following initial debulking surgery (27). Recently, Taliento et al. found that circulating tumor DNA was significantly associated with worse PFS and OS in patients with epithelial ovarian cancer (28). A recent review provides up-to-date evidence and summarizes the currently available therapeutic options for the treatment of ovarian cancer recurrence, investigating the factors that must be considered when choosing the best therapy, including molecular characterization and disease burden, while also presenting the limitations of current treatment options (29). Therefore, relying solely on BRCA mutation/HRD status as a clinical genetic indication for PARPi therapy is inadequate. Multiple factors—including clinical characteristics, tumor type and stage, quality of prior surgery, chemotherapy regimens, maintenance treatment protocols, biochemical markers, and pathological profiles—may influence PARPi efficacy and prognosis. Identifying key prognostic factors among a vast array of clinicopathological variables, eliminating redundancies, and constructing a robust and precise prediction model for PARPi efficacy and prognosis is complex. Traditional statistical approaches are often insufficient to address these complexities. Consequently, the integration of multimodal data using advanced machine learning techniques is essential. This approach promises to enhance the prediction of PARPi efficacy and refine prognostic assessments, thereby informing personalized treatment strategies.

**Figure 1:**
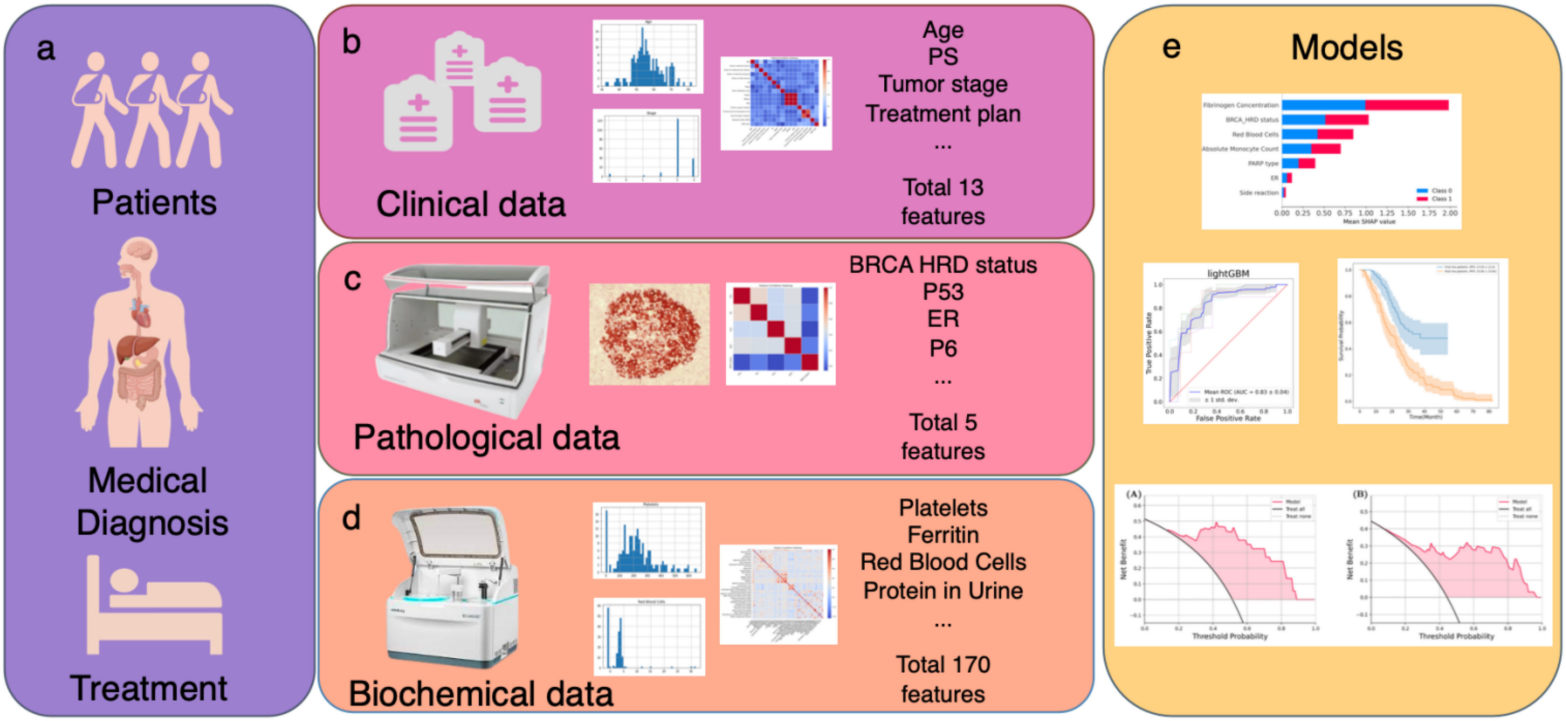
Schematic outline of the study. **(a–d)** Multiple data modalities were acquired through routine diagnosis to provide information for clinical decision making: **(b)** clinical information and treatment plan. **(c)** pathological information. **(d)** biochemical examination information. **(e)** Integrated multimodal analyses to construct efficacy and prognosis prediction model by PFS.

Machine learning (ML) has been widely applied in the medical field recently (30, 31). Given the vast amount of medical data, intricate patterns, and individual-specific expressions, ML offers unique advantages. It can extract significant patterns from complex medical datasets and achieve optimal model performance by identifying the most contributory feature combinations. ML has been extensively employed in cancer prognosis research, providing effective and accurate prognostic conclusions based on cancer sample data (32–34). In this work, we set out to investigate the clinical, pathological, and biochemical information obtained during routine diagnostic and therapeutic work in patients with ovarian cancer (**Figure 1a**). We performed correlation analysis and feature screening for the three types of features (**Figures 1b–d**). Finally, ML algorithms were used to construct a prognostic prediction model (**Figure 1e**).

## 2 Methods

### 2.1 Patients

This multi-center retrospective study analyzed data from 251 ovarian cancer patients collected between August 2018 and November 2023. The cohort comprised 215 cases from the initial single-center dataset at Hunan Cancer Hospital, supplemented by 26 cases from Xiangya Hospital of Central South University and 10 cases from The Second Xiangya Hospital of Central South University through subsequent multicenter collaboration. The inclusion criteria were as follows: histologically confirmed ovarian cancer; R0 residual tumor status; received platinum-based chemotherapy; received PARPi as maintenance therapy for more than 3 months. Exclusion criteria were as follows: follow-up time less than 6 months. This study was approved by the ethics committee of Hunan Cancer Hospital.

### 2.2 Data

Basic patient data were retrieved from the electronic medical record, and tumor recurrence and survival status were obtained by biweekly telephone follow-up. The dimensions of information collected included the patient’s clinical data (age, gender, performance status, height, weight, body mass index, marriage age, number of pregnancies, number of abortions, history of endocrine chronic diseases, history of cardiovascular diseases, history of infectious diseases, history of other cancers, pathological stage, pathological classification, metastasis type and platinum sensitive status); immunohistochemical data (P53, ER, P16, Ki67); biochemical data (blood routine, urine routine et al.); treatment data (treatment line, chemotherapy, PARPi type, toxicity and side effects, primary surgery hospital and secondary surgery hospital); BRCA mutation/HRD status; outcome data (patient recurrence status). Among the outcome measures, we agreed that a good outcome was defined as a PFS ≥ 24 months in primary ovarian cancer patients and a PFS ≥ 12 months in recurrent ovarian cancer patients. The classification into the primary or recurrent ovarian cancer patient cohort was determined based on the timing of the patients’ first use of PARPi. Patients were categorized into the primary cohort if their first use of PARPi occurred during the initial diagnosis of ovarian cancer, while those who first used PARPi during the recurrent phase were classified into the recurrent patient cohort.

### 2.3 Statistical analysis

In the exploration phase of data analysis, the Shapiro-Wilk test was used to test the normality of features, and the Spearman correlation coefficient was used to analyze the correlation between features. The Cox proportional hazards regression model is a statistical method used in survival analysis. We used the Cox proportional hazards model to perform univariate analysis for all variables. Variables that were significant with a P value of less than 0.05 in the Cox univariate analysis were entered into the subsequent Cox multivariate analysis. All the above analyses were implemented using Python3.6, where the Shapiro-Wilk test is from the Scipy library (35), the Spearman correlation coefficient analysis was performed using Pandas library (36), and Cox variable analysis was performed using Lifelines library (37).

### 2.4 Machine learning

#### 2.4.1 Feature selection

To ensure the objectivity and stability of feature selection, the following features screening strategy was conducted for clinical, pathological, and biochemical features:

##### 2.4.1.1 Remove features with a correlation value greater than 0.8

The correlation is calculated for every pair of features. For feature pairs with a correlation greater than 0.8, the occurrence frequency of each feature is counted, and the feature with the highest frequency is removed. The correlation among the remaining features is then recalculated, and this process is repeated until the correlation between all features is less than 0.8.

##### 2.4.1.2 Retain features with P values less than 0.05

Each feature was assessed by fitting a univariate Cox proportional hazards regression model using the Python Lifelines software package. The risk model was computed on the training set, and univariate coefficients and significance confidence were plotted. For features where the model failed to converge, the fit was retried using L2 regularization with a parameter c = 0.2. If the model still failed to converge, a P value of 1 and a hazard ratio (HR) of 1 were assigned. Features with P values less than 0.05 were retained for subsequent analysis.

##### 2.4.1.3 Exclude features with a VIF greater than 10

VIF is a statistical tool used to detect multicollinearity among features, which can lead to model instability and diminish both explanatory and predictive performance. VIF measures the linear relationship between each feature and the others by performing linear regression on each feature against the others, calculating the regression coefficient, and determining the R^2^ of the regression model. VIF is then calculated using the formula $\text{VIF}=\frac{1}{1-{\text{R}}^{2}}$ . Generally, VIF values greater than 10 indicate strong collinearity among features. During feature screening, features with high VIF values are eliminated to reduce the impact of multicollinearity.

##### 2.4.1.4 Preserve stable features

Elastic Net is a linear regression method that combines L1 regularization (Lasso) and L2 regularization (Ridge), which can be used for feature selection and model stability enhancement. When using the Elastic Net method for feature selection, the stability and reliability of selected features are assessed by observing changes in different data sets and model parameters. In this study, the scikit-learn library is used to implement feature selection for Elastic Net stability (38).

#### 2.4.2 Model construction

In the process of model construction, 7 common ML algorithms are selected to build the model (38, 39).

- Linear model: Logistic Regression(LR)
- Nearest neighbor method: K-Nearest Neighbors (KNN)
- Ensemble method: Random Forest (RF), lightGBM, and XGBoost
- Support Vector Machine: Support Vector Machine (SVM)
- Probabilistic model: Naive Bayes

In the data preprocessing stage, features with more than 50% missing data were deleted. Given that KNN and SVM algorithms are highly sensitive to data scale, Z-score normalization was applied for these models, while normalization was deemed unnecessary for other models.

#### 2.4.3 Model parameters tunning

The performance of a ML model is often influenced by its parameters, which are tuned to optimize the model’s performance and enhance its generalization capability. The primary objective of parameter tuning is to identify the optimal combination of parameters that can maximize or minimize performance metrics (such as accuracy, precision, recall, etc.), while preventing overfitting and improving the model’s ability to generalize. During the model tuning stage, the GridSearch method is employed to conduct a comprehensive parameter search (38).

#### 2.4.4 Model evaluation

The internal cohort comprised 215 ovarian cancer cases from Hunan Cancer Hospital (August 2018-November 2023), which were randomly partitioned into training and internal validation sets at a 4:1 ratio for model development. External validation was conducted using two datasets: 26 cases from Xiangya Hospital of Central South University and 10 cases from The Second Xiangya Hospital of Central South University, collectively forming a 36-case multicenter validation cohort.

In the model training and validation process, five-fold cross-validation was utilized. The dataset was randomly divided into five equal subsets. Each time, four subsets were used for training, and the remaining subset was used for testing. This process was repeated five times, and the average of the results was calculated to reduce the bias caused by different data partitions. The performance indicators selected were AUC, accuracy, F1 score, sensitivity, and specificity.

To assess the generalization ability of a ML model, a key step is to ensure that the model performs well on unseen data (40). However, numerous factors influence this ability, including model complexity, training set size, consistency and stability of data distribution between training and test sets, and the distribution state of the loss function in parameter space. Prior research has extensively examined model generalization across various models and application domains (40–43). Commonly employed techniques for evaluating generalization encompass comparing model performance on training and test sets, cross-validation, and multi-index evaluation. Discrepancies between performance on training and test sets may indicate overfitting or underfitting. Additionally, comprehensive evaluation entails consideration of multiple performance metrics such as accuracy, precision, recall, F1 score, and area under the ROC curve (AUC). While AUC serves as a primary metric for generalization evaluation, offering a holistic view of model performance across classification thresholds and robustness to class imbalance, it lacks specificity in assessing individual category performance, particularly for rare events. Moreover, AUC’s inability to directly address overfitting and underfitting limits its comprehensive assessment of model generalization.

To address the limitations of current generalization metrics, we propose the concept of Model Generalization Ability (MGA). MGA integrates the AUC metric on the test set (as the primary generalization metric) with the consistency of AUC between the training and test sets to evaluate both the stability and generalization ability of various ML models on a given dataset. We define MGA as:


$$MGA=AUC_{test\;set\;}\times R_{model\;generalization\;}$$

where AUC_test set_ is the AUC value of the model on the test set, and R_model generalization_ is the ratio of the test set AUC to the training set AUC, given by:


$$R_{model\;generalization}\;=\;\frac{AUC_{test\;set}}{AUC_{training\;set}}$$

A higher AUC_test set_ indicates that the model generalizes well to unseen data, which is often prioritized over training set performance when evaluating different models. This is because test set performance more accurately reflects the model’s true generalization ability. Conversely, a higher R_model generalization_ denotes greater consistency between the model’s performance on the training and test sets, signifying enhanced model stability. A model exhibiting both high AUC_test set_ and R_model generalization_ demonstrates superior generalization power, characterized by stable outputs and robust predictive performance on unfamiliar data. This dual consideration not only meets the requirements for evaluating generalization ability but also mitigates the issues of multiple comparisons inherent in multi-metric evaluations. By incorporating both performance and consistency, MGA provides a comprehensive assessment of a model’s generalization capabilities.

Finally, calibration curve analyses were utilized to compare the agreement between predicted probabilities and observed outcomes. Decision Curve Analysis (DCA) was performed to quantify the net benefits across different threshold probabilities, thereby assessing the clinical utility of the model and determining its effectiveness under various threshold probabilities.

#### 2.4.5 Model interpretation

To account for model features interpretability, we use SHapley Additive exPlanations(SHAP), which provides a systematic and unbiased approach to interpreting the predictions of ML models. The advantages of SHAP are as follows:

- Interpretability: SHAP enhances the interpretability of model predictions by assigning weights to each feature. This enables users to understand the model’s dependency on different features, thereby gaining more insight into the model’s decision-making process.
- Fairness: Based on Shapley values, SHAP ensures a fair contribution of each feature. This method appropriately weights each feature, preventing excessive emphasis on any single feature and helping to avoid bias or unfairness in model interpretation.
- Increase in trust: Understanding model predictions through SHAP can increase trust in the model. When users comprehend the reasons behind specific predictions, they are more likely to trust the model’s reliability, particularly in critical decision-making domains such as healthcare and finance.
- Problem diagnosis: SHAP facilitates the identification of model weaknesses and potential issues. By analyzing SHAP values, users can determine which features most significantly impact the model’s predictions, contributing to improvements in the model’s performance and robustness.

## 3 Results

### 3.1 Patients and disease characteristics

**Table 1** presents the distribution of clinical and pathological characteristics for the internal cohort of 215 patients, respectively. The mean age of the patients was 55.40 ± 8.73 years. Regarding prior medical conditions, 3.26% of the patients had chronic endocrine-related diseases, 13.95% had cardiovascular-related diseases, 1.86% had infectious diseases, and 8.37% had combined other cancers. At initial presentation, 65.12% of the patients had stage III tumors, and 22.79% had stage IV tumors, with 91.16% exhibiting metastases at diagnosis. 40.93% of the patients were primary ovarian cancer patients, and 59.07% of the patients were recurrent ovarian cancer patients. In the primary ovarian cancer patients, not all patients experienced recurrence, with 47.7% of the patients having already relapsed. Among the recurrent ovarian cancer patients, patients with their first recurrence accounted for 73.2%, while those with second or subsequent recurrences accounted for 27.8%. Taxol plus platinum (TP) regimen was administered to 40.93% of the patients, and TP plus bevacizumab to 50.70%. For maintenance therapy, olaparib was used in 56.28% of the patients, niraparib in 31.02%, and both olaparib and niraparib in 2.79%.

**Table 1:** Clinical characteristics of the patients (n=215). Categorical variables were expressed as Count(Percent), and continuous variables were expressed as Mean ± SD.

Characteristics	Statistics
Age	55.40 ± 8.73 years
Marriage age	23.19 ± 3.05 years
Height	1.56 ± 0.05 m
Weight	54.09 ± 8.03 kg
BMI	22.16 ± 3.08 kg/m^2^
Number of pregnancies	
0	4 (1.86)
1	34 (15.81)
2	53 (24.65)
>=3	103 (47.91)
Missing	21 (9.77)
Number of births	
0	7 (3.26)
1	81 (37.67)
2	81 (37.67)
>=3	25 (11.63)
Missing	21 (9.77)
PS	
0 score	194 (90.23)
1 score	18 (8.37)
2 score	3 (1.40)
Pathological type	
Serous	179 (83.26)
Mucinous	30 (13.95)
Clear cell	2 (0.93)
Endometrioid	4 (1.86)
Stage	
I	4 (1.86)
II	12 (5.58)
III	140 (65.12)
IV	49 (22.79)
Missing	10 (4.65)
Tumor metastasis type	
Without metastasis	19 (8.84)
Organ metastasis	66 (30.70)
Abdominal cavity, uterus or intestine metastasis	130 (60.47)
Primary surgery hospital	
Cancer Hospital	114 (53.02)
Non Cancer Hospital	101 (46.98)
Chronic endocrine history	
No	208 (96.74)
Yes	7 (3.26)
History of cardiovascular disease	
No	185 (86.05)
Yes	30 (13.95)
History of infectious diseases	
No	211 (98.14)
Yes	4 (1.86)
History of other tumors	
No	197 (91.63)
Yes	18 (8.37)
Platinum sensitive	
Yes	204 (94.88)
No	11 (5.12)
Treatment before PARP	
TP	88 (40.93)
TP + Bevacizumab	109 (50.7)
Others	18 (8.37)
PARPi type	
Olaparib	121 (56.28)
Niraparib	71 (33.02)
Olaparib + Niraparib	6 (2.79)
Fluzoparib	16 (7.44)
Missing	1 (0.47)
Treatment line	
Primary ovarian cancer	88 (40.93)
Recurrent ovarian cancer	127 (59.07)
Second Surgery Hospital	
Hunan Cancer Hospital	90 (41.86)
Others	29 (13.49)
Missing	96 (44.65)
BRCA mutation/HRD status	
No	102 (47.44)
Yes	113 (52.56)
P53	
No	30 (13.95)
Yes	112 (52.09)
Missing	73 (33.95)
ER	
Weak	72 (33.49)
Medium	50 (23.26)
Strong	14 (6.51)
Missing	79 (36.74)
P16	
No	9 (4.19)
Yes	91 (42.33)
Missing	115 (53.49)
Ki67	51.01 ± 23.14%

### 3.2 Statistical analysis

The mean PFS was 27.93 ± 11.00 months for primary ovarian cancer patients and 23.04 ± 15.65 months for recurrent ovarian cancer patients (**Supplementary Figure 1**). Significant differences were observed in the PFS distribution between primary and recurrent ovarian cancer patients (P < 0.001). Consequently, statistical analyses and predictive model construction were conducted separately for these patient cohorts and Cox proportional hazards regression analysis was performed for all characteristics. The results for primary and recurrent ovarian cancer patients were presented in **Table 2**. Only variables with P < 0.05 in the univariate Cox analysis were included in the tables. Among primary ovarian cancer patients, the variables significantly associated with PFS (P < 0.05) in the univariate analysis included BRCA mutation/HRD status, absolute value of lymphocytes (AVOL), PARPi type, antibody-ABO, total bile acids (TBAs), fibrinogen concentration, and thrombin time. While in the multivariate Cox analysis, only TBAs remained significant (P = 0.04). The hazard ratios (HRs) from both univariate and multivariate analyses indicate that BRCA mutation/HRD status is a protective factor; BRCA mutation/HRD positivity reduces the risk of recurrence, consistent with the conclusion of numerous current studies (10–12). Conversely, TBAs was identified as a risk factor in the multivariate analysis, with each unit increase in TBAs associated with a 10% increase in recurrence risk. In the cohort of recurrent ovarian cancer patients, significant variables (P < 0.05) in the univariate analysis included Ki67, isoenzymes of aspartate aminotransferase (IOAA), fasting blood glucose (FBG), glycated hemoglobin, uric acid, and CA-199. In the multivariate Cox analysis, only CA-199 remained significant (P < 0.01).

**Table 2:** Results of Cox univariate and multivariate analysis of primary(n=88) and recurrent(n=127) ovarian cancer patients respectively.

Patient category	Feature	Univariate		Multivariate	
		HR	P	HR	P
Primary ovarian cancer patients	PARPi type	1.38 (1.02, 1.86)	0.04	1.3 0.92, 1.83)	0.13
	AVOL^a^	0.74 (0.56, 1.0)	0.05	0.87 (0.63, 1.2)	0.39
	Antibody-ABO^b^	1.0 (1.0, 1.0)	0.02	1.0 (1.0, 1.0)	0.07
	TBAs^c^	1.09 (1.02, 1.18)	0.02	1.10 (1.0, 1.2)	0.04
	Thrombin time^d^	1.05 (1.0, 1.09)	0.03	0.98 (0.91, 1.06)	0.69
	Fibrinogen concentration^e^	1.16 (1.05, 1.29)	<0.01	1.16(0.98, 1.38)	0.08
	BRCA mutation/HRD status	0.49 (0.26, 0.89)	0.02	0.75 (0.38, 1.50)	0.42
Recurrent ovarian cancer patients	Ki67	1.36 (1.05, 1.75)	0.02	1.27 (0.98, 1.65)	0.07
	IOAA^f^	1.01 (1.0, 1.02)	<0.01	1.01 (1.0, 1.02)	0.07
	FBG^g^	1.13 (1.04, 1.23)	<0.01	1.05 (0.95, 1.16)	0.37
	Glycated Hemoglobin^h^	1.12 (1.05, 1.20)	<0.01	1.07 (0.99, 1.15)	0.11
	Uric Acid^i^	1.0 (1.0, 1.0)	<0.01	1.0 (1.0, 1.0)	0.16
	CA-199^j^	1.0 (1.0, 1.01)	<0.01	1.0 (1.0, 1.01)	<0.01

a Blood routine: Absolute value of lymphocytes.

b ABO blood group: specific antibody-ABO blood type.

c Routine liver function tests: Total Bile Acids.

d Blood clotting routine four items: Thrombin time.

e Blood clotting routine four items: fibrinogen concentration.

f Routine liver function tests: Isoenzymes of Aspartate Aminotransferase.

g Fasting blood glucose: Fasting blood glucose.

h Determination of hemoglobin A1c: Glycated Hemoglobin.

i Routine renal function program: Uric Acid.

j Carbohydrate antigen-199 (CA-199): Carbohydrate antigen-199.

### 3.3 Machine learning

We employed ML to construct PARPi response and prognosis prediction models in ovarian cancer, consisting of two main stages: feature selection and model construction/prediction. During the feature selection stage, correlation analysis was performed for all features. Features were screened based on the principle of excluding highly correlated features (**Supplementary Figures 2**, **3**). **Supplementary Tables 1**, **2** present the features with P < 0.05 following Cox univariate analysis. Subsequently, VIF values and elastic net feature stability values were calculated. Features with VIF >= 10 and stability <= 0.7 were excluded. For primary ovarian cancer patients, the final model included the following features: BRCA mutation/HRD status, PARPi type, antibody-ABO, TBAs, fibrinogen concentration, and thrombin time. For recurrent ovarian cancer patients, the final model incorporated Ki67, IOAA, FBG, glycated hemoglobin, uric acid, and CA-199.

**Figure 2:**
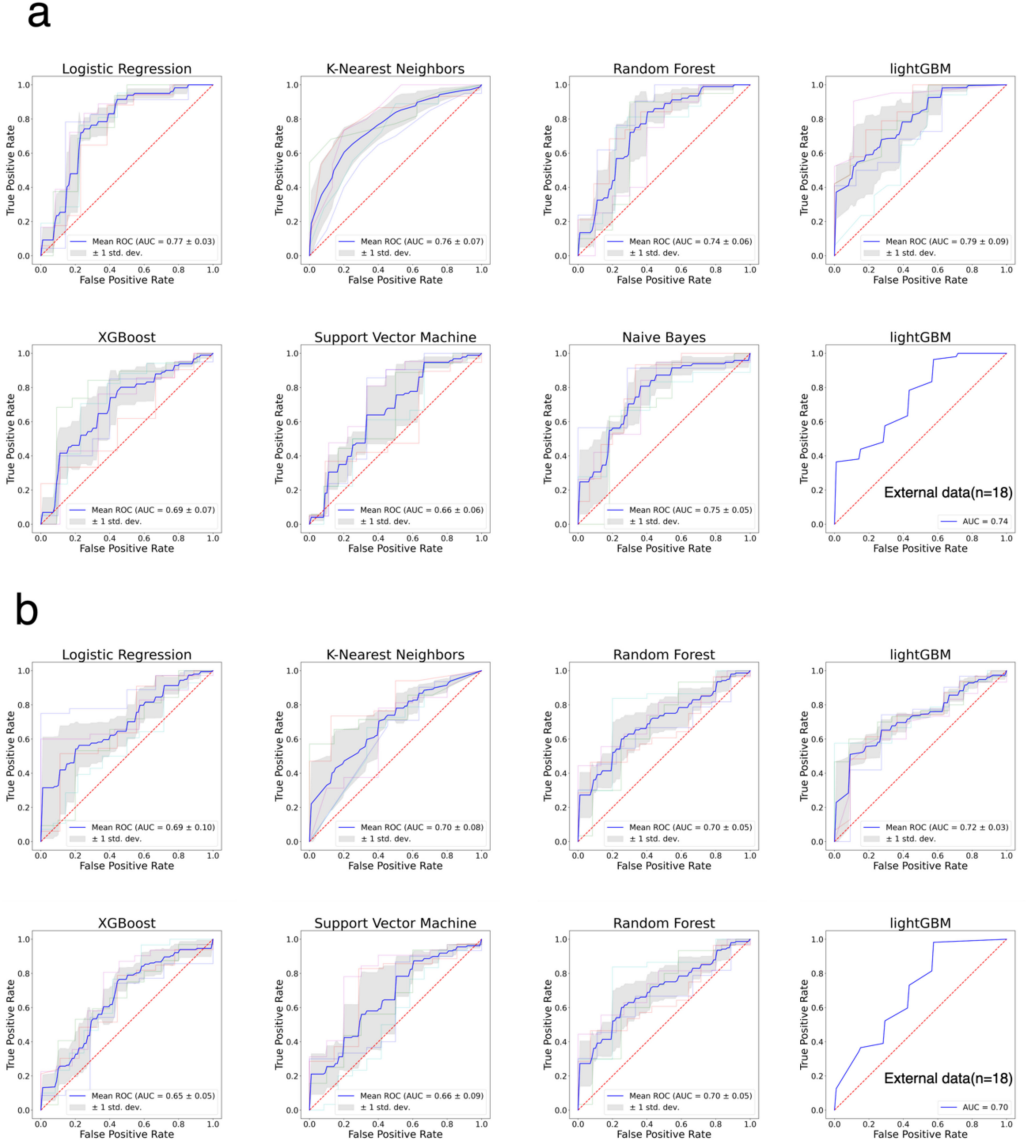
The ROC curves for the classification of patients with good and bad PFS patients. **(a)** ROC curves of seven models for primary ovarian cancer patients. **(b)** ROC curves of seven models for recurrent ovarian cancer patients.

We selected seven ML models—LR, KNN, RF, LightGBM, XGBoost, SVM, and Naive Bayes—to construct prediction models for both primary and recurrent ovarian cancer patient data. Optimal parameter combinations for each model were identified through grid search, and model evaluation was conducted using 5-fold cross-validation. **Figure 2** illustrates the ROC curves for each of the seven models on internal and external datasets for primary and recurrent ovarian cancer patients. **Table 3** presents a comparison of various performance metrics for these models on primary and recurrent ovarian cancer patients, respectively. For primary ovarian cancer patients, the best-performing model according to the MGA was LightGBM, with an MGA of 0.77 ± 0.19. The performance metrics for LightGBM in internal test set were: AUC = 0.79 ± 0.09, accuracy = 0.61 ± 0.10, sensitivity = 0.65 ± 0.19, specificity = 0.63 ± 0.27, F1 score = 0.68 ± 0.10, precision = 0.78 ± 0.16, and recall = 0.65 ± 0.19. For recurrent ovarian cancer patients, LightGBM also demonstrated the best performance, with an MGA of 0.76 ± 0.05. The performance metrics for this model in internal test set were: AUC = 0.72 ± 0.03, accuracy = 0.60 ± 0.11, sensitivity = 0.55 ± 0.22, specificity = 0.74 ± 0.28, F1 score = 0.65 ± 0.14, precision = 0.90 ± 0.07, and recall = 0.55 ± 0.22. Furthermore, rigorous external validation conducted in independent multicenter cohorts confirmed the robustness of the LightGBM model, demonstrating discriminative performance with AUC values of 0.74 in the primary cohort and 0.70 in the recurrent cohort. Complete metrics including sensitivity, specificity, and clinical utility metrics are systematically documented in **Table 3**.

**Table 3:** Summary of ML algorithms predictive performance for the primary and recurrent ovarian cancer patients(Mean ± SD).

Patient category	Dataset	models	AUC	Accuracy	Sensitivity	Specificity	F1 score	Precision	Recall	MGA
Primary ovarian cancer patients	Internal	LR	0.77 ± 0.03	0.69 ± 0.07	0.73 ± 0.15	0.61 ± 0.18	0.74 ± 0.08	0.78 ± 0.06	0.73 ± 0.15	0.77 ± 0.09
		KNN	0.76 ± 0.07	0.62 ± 0.08	0.54 ± 0.16	0.73 ± 0.08	0.61 ± 0.14	0.72 ± 0.09	0.54 ± 0.16	0.71 ± 0.14
		RF	0.74 ± 0.06	0.74 ± 0.03	0.89 ± 0.03	0.42 ± 0.07	0.82 ± 0.02	0.76 ± 0.04	0.89 ± 0.03	0.60 ± 0.11
		SVM	0.66 ± 0.06	0.61 ± 0.06	0.6 ± 0.18	0.65 ± 0.21	0.65 ± 0.1	0.78 ± 0.08	0.6 ± 0.18	0.44 ± 0.08
		lightGBM	0.79 ± 0.09	0.61 ± 0.1	0.65 ± 0.19	0.63 ± 0.27	0.68 ± 0.1	0.78 ± 0.16	0.65 ± 0.19	0.77 ± 0.19
		XGBoost	0.69 ± 0.07	0.63 ± 0.09	0.71 ± 0.24	0.57 ± 0.22	0.67 ± 0.15	0.74 ± 0.14	0.71 ± 0.24	0.48 ± 0.09
		Naive Bayes	0.75 ± 0.05	0.75 ± 0.03	0.77 ± 0.12	0.67 ± 0.19	0.78 ± 0.06	0.81 ± 0.04	0.77 ± 0.12	0.72 ± 0.12
	External	lightGBM	0.74	0.62	0.62	0.63	0.61	0.77	0.62	–
Recurrent ovarian cancer patients	Internal	LR	0.69 ± 0.1	0.68 ± 0.15	0.73 ± 0.26	0.54 ± 0.27	0.75 ± 0.18	0.86 ± 0.08	0.73 ± 0.26	0.68 ± 0.24
		KNN	0.7 ± 0.08	0.6 ± 0.07	0.59 ± 0.11	0.69 ± 0.18	0.69 ± 0.07	0.86 ± 0.08	0.59 ± 0.11	0.49 ± 0.11
		RF	0.7 ± 0.05	0.55 ± 0.08	0.42 ± 0.16	0.86 ± 0.13	0.55 ± 0.16	0.88 ± 0.08	0.42 ± 0.16	0.49 ± 0.07
		SVM	0.66 ± 0.09	0.68 ± 0.08	0.71 ± 0.15	0.62 ± 0.19	0.76 ± 0.08	0.84 ± 0.08	0.71 ± 0.15	0.46 ± 0.13
		lightGBM	0.72 ± 0.03	0.6 ± 0.11	0.55 ± 0.22	0.74 ± 0.28	0.65 ± 0.14	0.9 ± 0.07	0.55 ± 0.22	0.76 ± 0.05
		XGBoost	0.65 ± 0.05	0.57 ± 0.07	0.46 ± 0.13	0.84 ± 0.06	0.6 ± 0.12	0.88 ± 0.05	0.46 ± 0.13	0.43 ± 0.06
		Naive Bayes	0.73 ± 0.08	0.63 ± 0.1	0.62 ± 0.18	0.65 ± 0.19	0.7 ± 0.13	0.86 ± 0.06	0.62 ± 0.18	0.75 ± 0.15
	External	lightGBM	0.70	0.61	0.50	0.67	0.71	0.82	0.60	–

**Figure 3:**
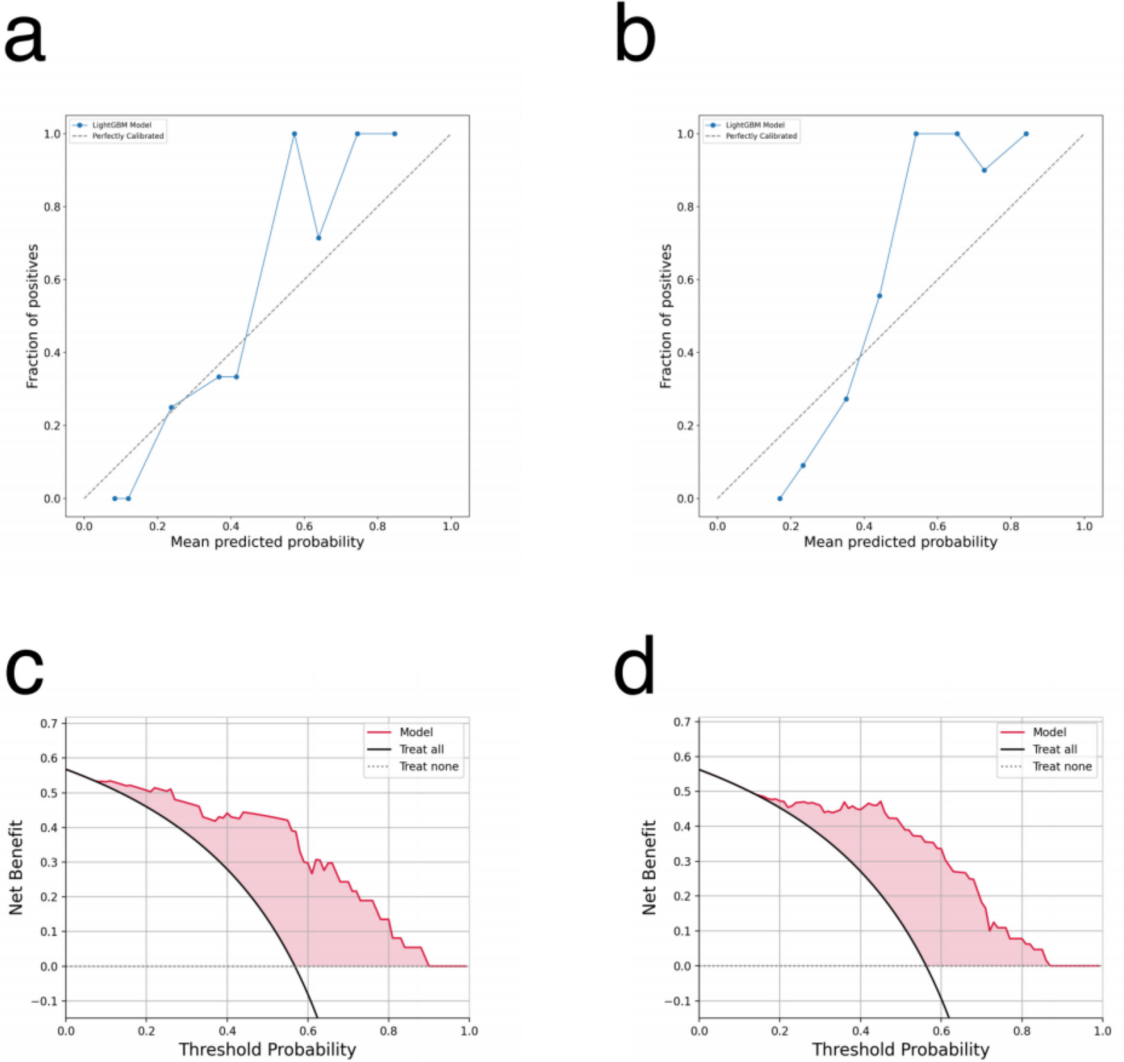
**(a, b)** The calibration curve for the lightGBM model: **(a)** primary ovarian cancer patients. **(b)** recurrent ovarian cancer patients. **(c, d)** The DCA curve for the lightGBM model: **(c)** primary ovarian cancer patients. **(d)** recurrent ovarian cancer patients.

The calibration curves for both the primary and recurrent ovarian cancer patient models are presented in **Figures 3a, b**. These curves evaluate the reliability of probability predictions by comparing the model-predicted probabilities (x-axis) with the observed positive rates (y-axis). The analysis reveals that the solid blue line (model predictions) closely approximates the dashed line (perfect calibration) across most intervals (e.g., 0.1–0.5), indicating accurate efficacy predictions for low-to-moderate probability ranges. However, in the high-probability interval (>0.6), the observed positive rates are slightly higher than the predicted probabilities, suggesting a potential mild underestimation of efficacy for high-probability samples. This finding underscores the importance of avoiding overreliance on a single high-probability threshold for clinical decision-making. The DCA curves for the primary and recurrent ovarian cancer patient models are illustrated in **Figures 3c, d**. These curves assess the clinical utility of the models across various decision-making scenarios by analyzing the relationship between threshold probabilities and net benefit. The red curve (model) demonstrates significantly higher net benefit than the black line (“Treat all”) and gray line (“Treat none”) across most threshold probability ranges. This indicates that employing the model to guide PARP inhibitor treatment could substantially enhance clinical net benefit within these intervals.

**Figure 4:**
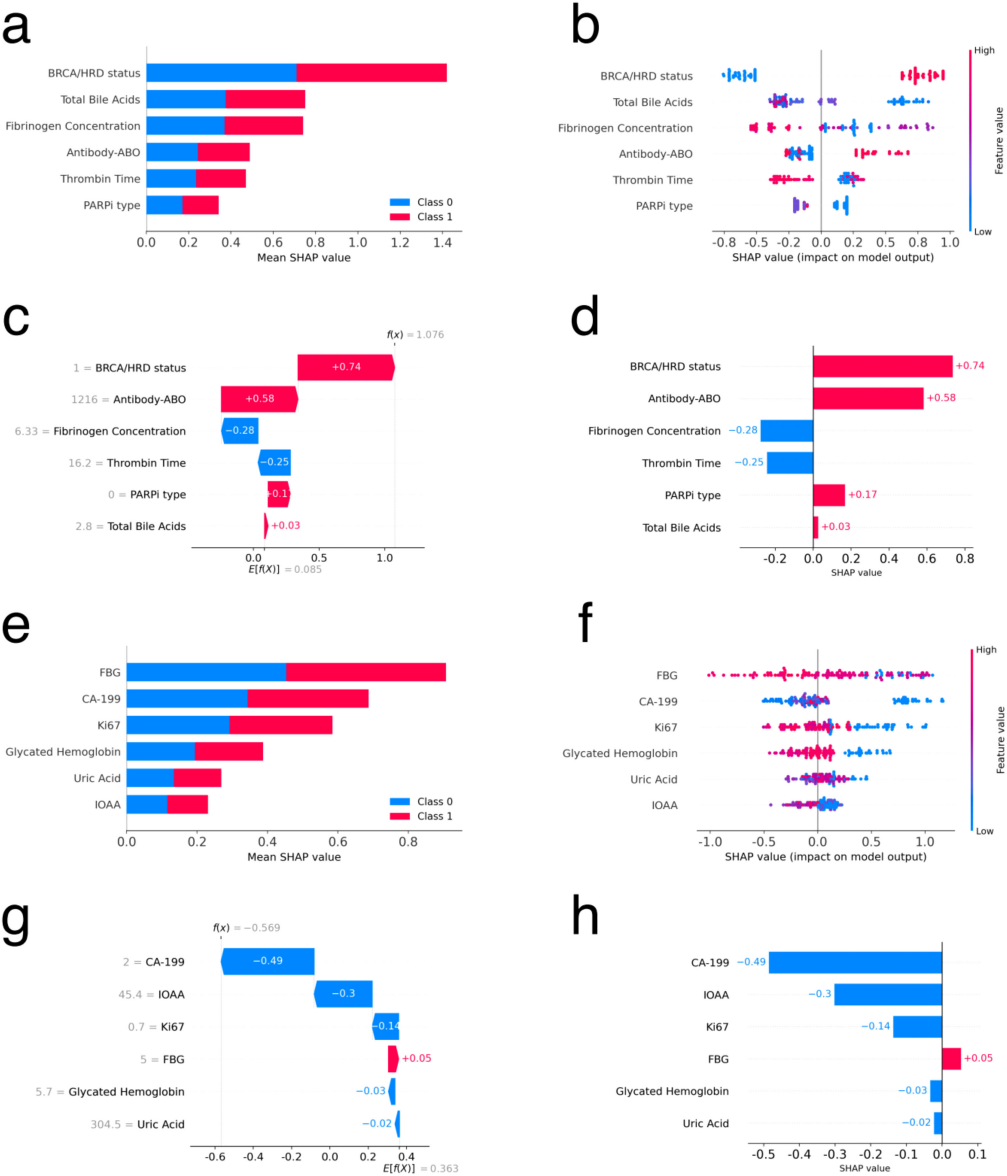
Feature interpretation using SHAP for primary and recurrent ovarian cancer patients. **(a, e)** Bar plot of feature importance sorted by mean SHAP value for primary and recurrent ovarian cancer patients respectively. **(b, f)** Density scatter plot of SHAP values for each feature for primary and recurrent ovarian cancer patients respectively. **(c, g)** Local waterfall plot of feature importance sorted by mean SHAP value for one sample for primary and recurrent ovarian cancer patients respectively. **(d, h)** Local bar plot of feature importance sorted by mean SHAP value for one sample for primary and recurrent ovarian cancer patients respectively.

**Figures 4a–d** presents the results of interpreting the LightGBM model for primary ovarian cancer patients using SHAP. **Figures 4a, b** are global bar and scatter plots, respectively. **Figure 4a** illustrates the importance and rank of each feature’s contribution to the model, with BRCA mutation/HRD status, TBAs, and fibrinogen concentration ranking as the top three most important features. **Figure 4b** shows how each sample contributes to the model based on each feature. As depicted, samples with positive BRCA mutation/HRD status (value 1, represented by red dots) generally contribute positively to the model, indicating a tendency towards lower relapse risk. In contrast, samples with negative BRCA mutation/HRD status (value 0, represented by blue dots) typically contribute negatively, indicating a higher tendency towards relapse. Additionally, higher values of TBAs and fibrinogen concentration contributed negatively to the model. Patients treated with olaparib (value 0 for PARPi type) generally contributed positively to the model, while other PARPi types contributed negatively. **Figures 4c, d** depict the contribution of each feature to the model for a single sample. For this particular sample, a BRCA mutation/HRD status of 1 provided a positive contribution of 0.74 to the model. A TBAs value of 2.8 µmol/L (within the normal reference range of 0.5-10 µmol/L) contributed positively with a value of 0.03. Conversely, a fibrinogen concentration of 6.33 g/L (exceeding the normal reference range of 1.8-3.5 g/L) contributed negatively with a value of 0.28. These contributions are consistent with the overall trends observed in the global analysis.

**Figures 4e–h** presents the results of interpreting the LightGBM model using SHAP for recurrent ovarian cancer patients. **Figures 4e, f** are global bar and scatter plots, respectively. **Figure 4e** shows the importance and rank of each feature’s contribution to the model, with the top three features being FBG, CA-199, and Ki67. **Figure 4f** illustrates the contributions of all samples to the model based on each feature. The figure indicates a complex contribution trend, with no clear boundary to distinguish the impact of sample values on the model. However, in general, higher values of FBG, CA-199, Ki67, and glycated hemoglobin exhibit a negative trend in their contributions to the model. **Figures 4g**, **h** depict the contribution of each feature to the model for a single sample. These figures help in understanding how individual features influence the model’s predictions for specific cases.

## 4 Discussion

Ovarian cancer is widely recognized for its significant genomic disruption and high mutation rate (44). Clinical trials have demonstrated that patients with BRCA mutation or HRD can significantly benefit from PARPi (10–12). Interestingly, some non-HRD or BRCA-wild type patients may also experience benefits from PARPi (14–16). In the era of precision medicine, it is crucial to identify patients who will benefit from PARPi therapy, as from preoperative evaluation to postoperative treatment plans, personalized approaches for patients are receiving increasing attention (45, 46). At present, the prognostic factors influencing the efficacy of PARPi in ovarian cancer remain unclear. While clinical examinations, pathological and biochemical tests are readily available in practice, their combined predictive power is yet to be fully determined. In this study, we explored the impact of 188 potential influencing factors on PFS, including clinical, pathological, and biochemical information. Although classical statistical methods can identify associations between variables and outcomes (47), ML methods are more suitable for multivariate predictive classification tasks.

In this study, we collected data from 251 patients, encompassing clinical characteristics, pathological, and biochemical information, resulting in a total of 188 features. Among primary ovarian cancer patients, TBAs was identified as a significant prognostic factor for PFS through both Cox univariate (P = 0.02) and multivariate (P = 0.04) analyses. In the feature importance analysis of the ML model, the top three factors were BRCA mutation/HRD status, TBAs, and fibrinogen concentration. BRCA mutation/HRD status notably influences the prognosis of ovarian cancer. Consistent with many current studies, BRCA mutation/HRD-positive patients in our study tended to have better prognoses (10–12, 14, 15). TBAs, which are cholesterol-derived sterols and signaling molecules, play a key role in regulating cancer cell behavior through receptor-mediated functions. The activation of receptors such as liver X receptor, pregnane X receptor, vitamin D receptor, or constitutive androstane receptor has been shown to protect against ovarian cancer (21–23, 25). These protective effects, similar to those elicited by bile acids(BAs), include the inhibition of proliferation, invasion, epithelial-mesenchymal transition, *de novo* fatty acid biosynthesis, and the proportion of the cancer stem cell population, as well as improving the efficacy of chemotherapy (48–51). Additionally, BAs can influence the expression and activity of various PARP enzymes (24), and deoxycholic acid can regulate the expression of BRCA1 and estrogen receptors, thereby controlling the drug sensitivity of ovarian cancer cells (52). Thus, BAs may modulate the efficacy of PARPi.

CA-199 was identified as a significant prognostic factor for PFS in recurrent ovarian cancer through both Cox univariate (P < 0.01) and multivariate (P < 0.01) analyses. In the feature importance analysis of the ML model, the top three factors were FBG, CA-199, and Ki67. Our study findings from both Cox factor analysis and the ML model suggested that CA-199 and FBG were risk factors affecting prognosis, with increases in CA-199 and FBG levels associated with an elevated risk of poor prognosis. CA-199 is associated with primary cancers of the gastrointestinal system and ovary, serving as a diagnostic marker for gastrointestinal and ovarian mucoid cancers (53, 54). Zhu et al. conducted a study analyzing serum CA-199 levels in patients with normal postoperative serum CA-125 levels, confirming that an increase in postoperative serum CA-199 level was an independent risk factor for PFS and OS. Patients with elevated serum CA-199 levels exhibited significantly lower 5-year PFS and OS rates compared to those with normal levels (27). Furthermore, postoperative serum CA-199 levels have been shown to help identify subgroups of patients with normal postoperative CA-125 levels who are at higher risk of recurrence and death.

Additionally, the FBG levels of patients may serve as an important prognostic indicator. In ovarian tumors, increased expression of the transmembrane protein glucose transporter 1, responsible for glucose uptake, has been associated with shortened survival in ovarian cancer patients. Lamkin et al. found in univariate analysis that higher blood glucose levels were associated with shorter survival times (hazard ratio = 1.88; P < 0.05). Multivariate analysis adjusted for staging revealed that higher blood glucose levels were associated with shorter survival times (hazard ratio = 2.01; P = 0.04) and disease-free interval (hazard ratio = 2.32; P < 0.05). These findings suggest the prognostic value of blood glucose levels in ovarian cancer (26). In 2019, elevated fasting blood glucose was identified as the highest risk factor for ovarian cancer deaths, with a global increase in age-standardized mortality rates attributed to elevated fasting blood glucose across all Socio-demographic Index quintiles. This trend may be related to diabetes comorbidity-related deaths (55). Currently, diabetes has been confirmed as an independent risk factor for ovarian cancer mortality (56), and elevated blood glucose levels in ovarian cancer patients are predictive factors for poorer survival rates (26). Rapidly proliferating cancer cells benefit from the nutrient-rich microenvironment of high blood glucose levels, as they have increased metabolic demands and utilize glucose faster than healthy cells (57). Chronic hyperglycemia can increase oxidative stress, reduce the tumor-suppressive activity of adenosine monophosphate-activated protein kinase, impair the diversion capacity of hexosamine monophosphate, and lead to the accumulation of advanced glycation end products, affecting the mitogen-activated protein kinase NF-KB pathway (58). Angiogenesis is crucial for the growth of ovarian cancer, as solid tumors typically require the formation of new blood vessels to grow larger than 1-2mm (59). The expression of vascular endothelial growth factor(VEGF) has been found to be correlated with blood glucose levels, and VEGF is now recognized as an effective pro-angiogenic factor, suggesting that patients with uncontrolled diabetes may be more susceptible to ovarian cancer.

**Figure 5:**
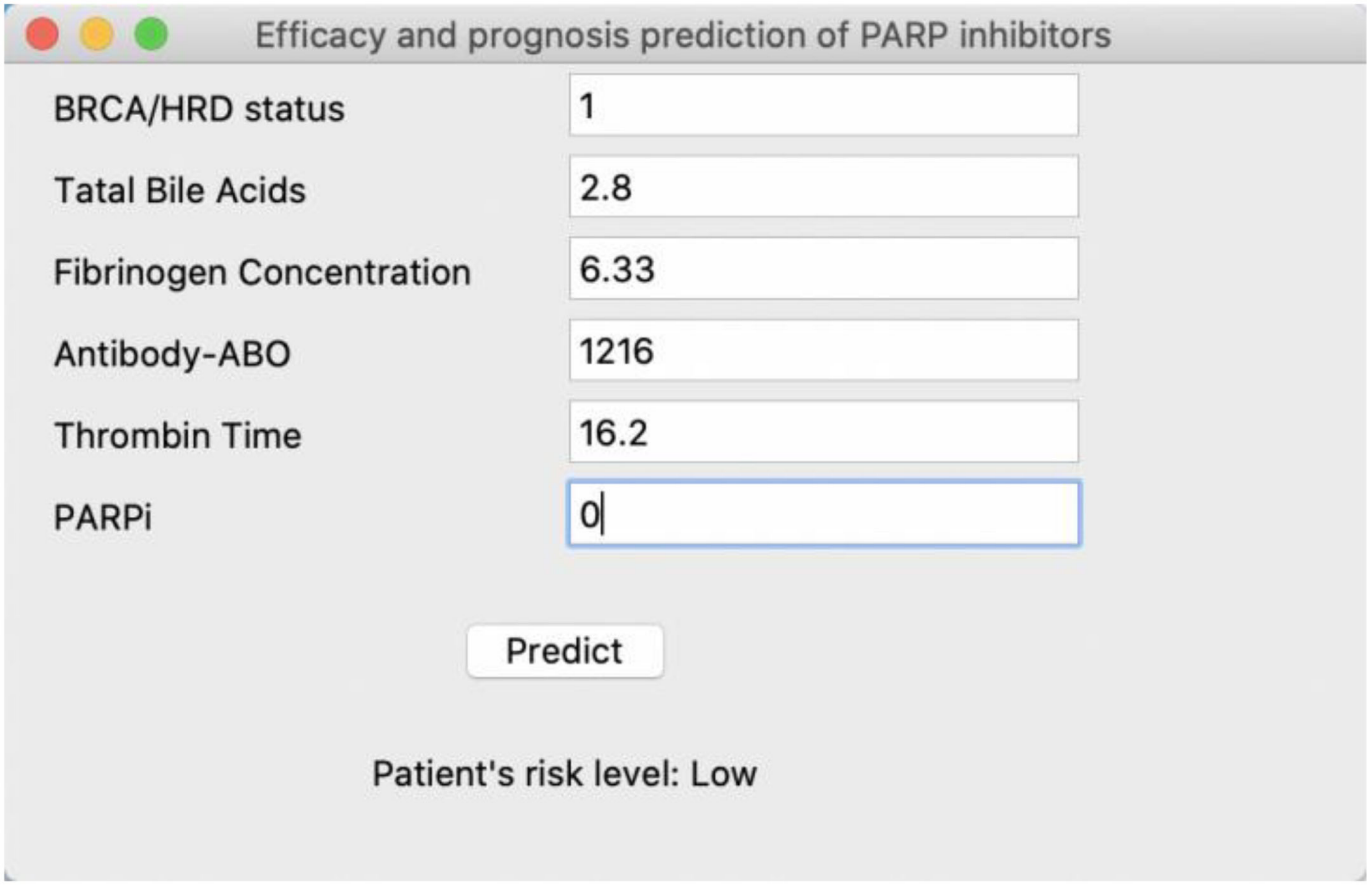
Interactive interface of the PARP inhibitor efficacy and prognosis prediction model for clinical use. Taking the PARP inhibitor efficacy and prognosis prediction model of primary ovarian cancer patient as an example, further details can be found at the GitHub link.

In this study, we employed ML techniques to construct separate efficacy prognosis prediction models for primary and recurrent ovarian cancer patients based on multimodal data. Both models demonstrated favorable performance, with AUC values of 0.79 ± 0.09 and 0.72 ± 0.03, respectively. Furthermore, we identified the top three factors with the greatest impact on the models: for primary ovarian cancer patients, these were BRCA mutation/HRD status, TBAs, and fibrinogen concentration, while for recurrent ovarian cancer patients, they were FBG, CA-199, and Ki67. Considering the interpretability limitations of ML models, we conducted feature analysis using SHAP. In primary ovarian cancer patients, BRCA mutation/HRD status positivity contributed positively to the model, while higher values of TBAs and fibrinogen concentration had a negative impact on the model. Patients treated with olaparib showed a general positive contribution to the model, whereas other PARPi types exhibited a negative contribution. In recurrent ovarian cancer patients, higher values of FBG, CA-199, Ki67, and glycated hemoglobin trended towards negative contributions to the model overall. Machine learning models often encounter challenges in feature interpretation and application. To address this, we have developed a user-friendly interface tool, as shown in **Figure 5**. This tool assists clinicians in inputting the necessary variables for the model and provides the patient’s risk level, offering guidance for treatment strategy. The tool can be easily deployed and run locally, greatly simplifying its use in clinical settings. For detailed code, deployment, and application instructions, please refer to the GitHub link: https://github.com/xiongxa/PARP_efficacy_prediction.

It should be noted that we intentionally explored data generated during standard treatment. Using these data instead of data specifically collected for computational modeling significantly reduces the adoption cost of the final model in clinical workflows, but these data were not collected specifically for modeling purposes. For most patients, we included BRCA gene mutation status but not HRD status, as complete HRD gene sequencing data was not available for all cases. While BRCA mutations are the first and most widely used genotype prognostic factor for PARPi efficacy in ovarian cancer, they are not sufficient to predict the efficacy of PARPi. Current research findings suggest that HRD-positive status is an important prognostic factor for PARPi. Based on synthetic lethality mechanisms, HRD is more widely distributed in ovarian cancer than BRCA mutations. This is because HRD can be caused not only by deleterious BRCA mutations but also by genomic alterations or epigenetic inactivation of BRCA genes and other defects independent of BRCA (60, 61), and it is associated with the efficacy of PARPi (62, 63). Therefore, it is hoped that more complete HRD gene sequencing data can be obtained in the future.

These results may provide research directions for exploring effective and accurate prognostic factors for PARPi efficacy in ovarian cancer. Therefore, there is an urgent need for large-scale, prospective clinical studies to explore effective and accurate prognostic factors for PARPi efficacy, thereby facilitating personalized PARPi treatment and expanding the use of PARPi to a more suitable population of ovarian cancer patients.

## Data Availability

The raw data supporting the conclusions of this article will be made available by the authors, without undue reservation.

## Supplementary material

Supplementary Figure 1 The PFS survival curve of primary and recurrent ovarian cancer patients. The blue and yellow line with the error bar shows the results of primary and recurrent ovarian cancer respectively.

Supplementary Figure 2 The Spearman correlation of primary ovarian cancer patients. **(a)** Spearman correlation between patient clinical characteristics; **(b)** Spearman correlation between pathological features of patients; **(c)** Spearman correlation between patient biochemical omics features; All three figures are symmetric along the diagonal.

Supplementary Figure 3 The Spearman correlation of recurrent ovarian cancer patients. **(a)** Spearman correlation between patient clinical characteristics; **(b)** Spearman correlation between pathological features of patients; **(c)** Spearman correlation between patient biochemical omics features; All three figures are symmetric along the diagonal.

Supplementary Table 1 Characteristic VIF and stability results in groups of primary ovarian cancer patients.

Supplementary Table 2 Characteristic VIF and stability results in groups of recurrent ovarian cancer patients.
