# Supplementary figures and images for: Multimodal data integration with machine learning for predicting PARP inhibitor efficacy and prognosis in ovarian cancer
Source: Front Oncol. 2025 Jun 4;15:1571193. doi: 10.3389/fonc.2025.1571193 (PMC12173870; doi:10.3389/fonc.2025.1571193)

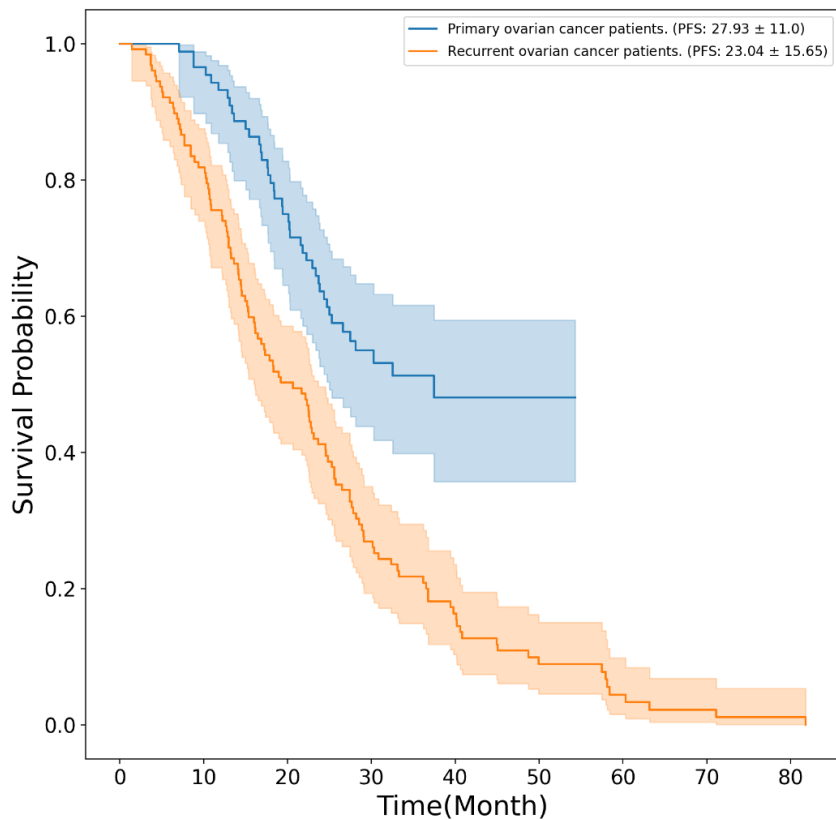

Supplement: Supplementary Figure 1 — The PFS survival curve of primary and recurrent ovarian cancer patients. The blue and yellow line with the error bar shows the results of primary and recurrent ovarian cancer respectively. [file Image1.pdf]

a

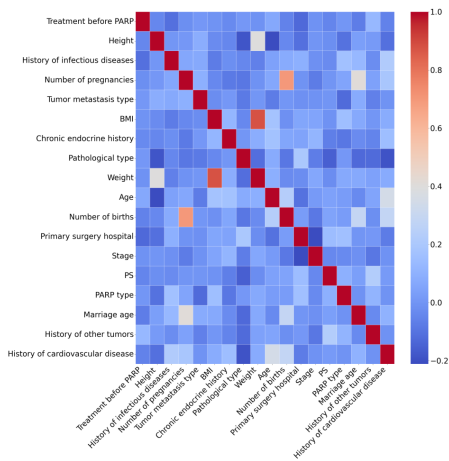

b

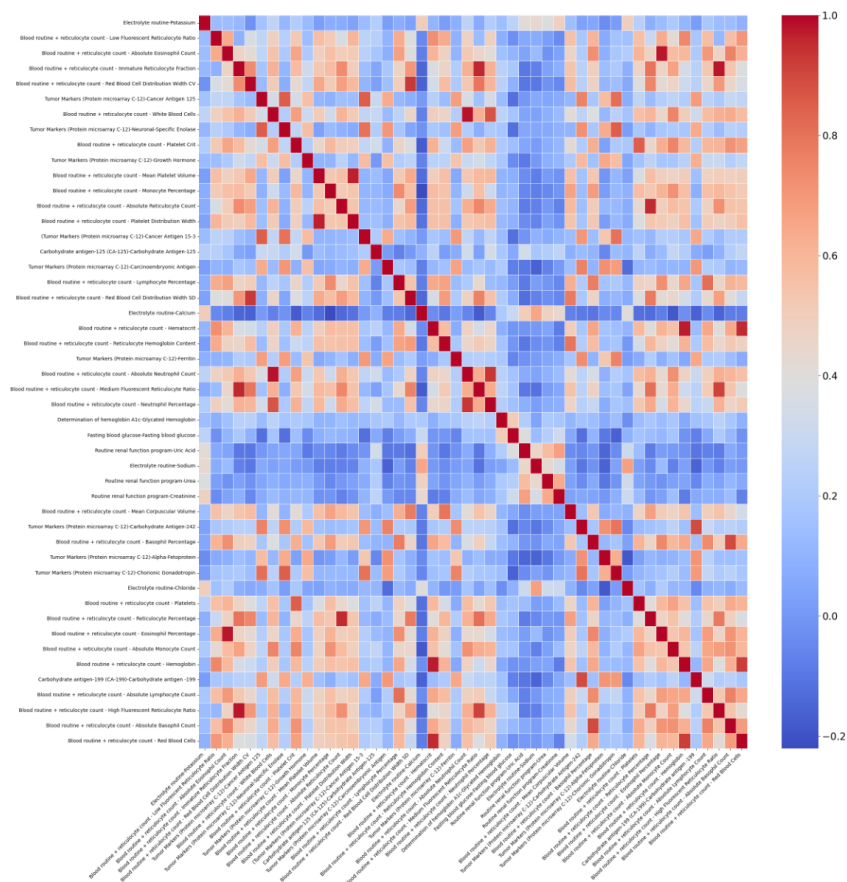

c

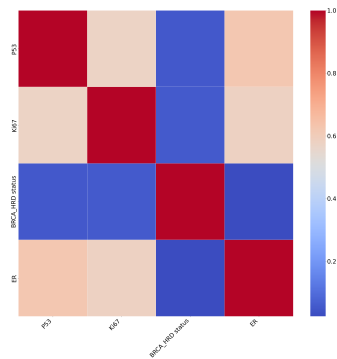

Supplement: Supplementary Figure 3 — The Spearman correlation of recurrent ovarian cancer patients. (a) Spearman correlation between patient clinical characteristics; (b) Spearman correlation between pathological features of patients; (c) Spearman correlation between patient biochemical omics features; All three figures are symmetric along the diagonal. [file Image3.pdf]
