# Supplementary material for: Multimodal data integration with machine learning for predicting PARP inhibitor efficacy and prognosis in ovarian cancer
Source: Front Oncol. 2025 Jun 4;15:1571193. doi: 10.3389/fonc.2025.1571193 (PMC12173870; doi:10.3389/fonc.2025.1571193)
Supplement: Supplementary Table 1 — Characteristic VIF and stability results in groups of primary ovarian cancer patients. [file Table1.docx]

Supplementary Table 1. Characteristic VIF and stability results in groups of primary ovarian cancer patients.

| Variable | VIF | Feature stability | Final selected variables |
| --- | --- | --- | --- |
| PARPi type | 1.14 | 1.0 | True |
| AVOL | 1.2 | 0.7 | False |
| Antibody-ABO | 1.12 | 0.8 | True |
| TBAs | 1.1 | 1.0 | True |
| Thrombin time | 3.74 | 1.0 | True |
| Fibrinogen concentration | 3.72 | 1.0 | True |
| BRCA mutation/HRD status | 1.16 | 1.0 | True |
