# Supplementary material for: Multimodal data integration with machine learning for predicting PARP inhibitor efficacy and prognosis in ovarian cancer
Source: Front Oncol. 2025 Jun 4;15:1571193. doi: 10.3389/fonc.2025.1571193 (PMC12173870; doi:10.3389/fonc.2025.1571193)
Supplement: Supplementary Table 2 — Characteristic VIF and stability results in groups of recurrent ovarian cancer patients. [file Table2.docx]

Supplementary Table 2. Characteristic VIF and stability results in groups of recurrent ovarian cancer patients.

| Variable | VIF | Feature stability | Final selected variables |
| --- | --- | --- | --- |
| Ki67 | 1.06 | 1.0 | True |
| IOAA | 1.18 | 1.0 | True |
| FBG | 1.74 | 1.0 | True |
| Glycated Hemoglobin | 1.66 | 1.0 | True |
| Uric Acid | 1.3 | 1.0 | True |
| CA-199 | 1.02 | 0.9 | True |
