# Supplementary material for: Multimodal data integration with machine learning for predicting PARP inhibitor efficacy and prognosis in ovarian cancer
Source: Front Oncol. 2025 Jun 4;15:1571193. doi: 10.3389/fonc.2025.1571193 (PMC12173870; doi:10.3389/fonc.2025.1571193)
Supplement: Supplementary Figure 2 — The Spearman correlation of primary ovarian cancer patients. (a) Spearman correlation between patient clinical characteristics; (b) Spearman correlation between pathological features of patients; (c) Spearman correlation between patient biochemical omics features; All three figures are symmetric along the diagonal. [file Image2.pdf]

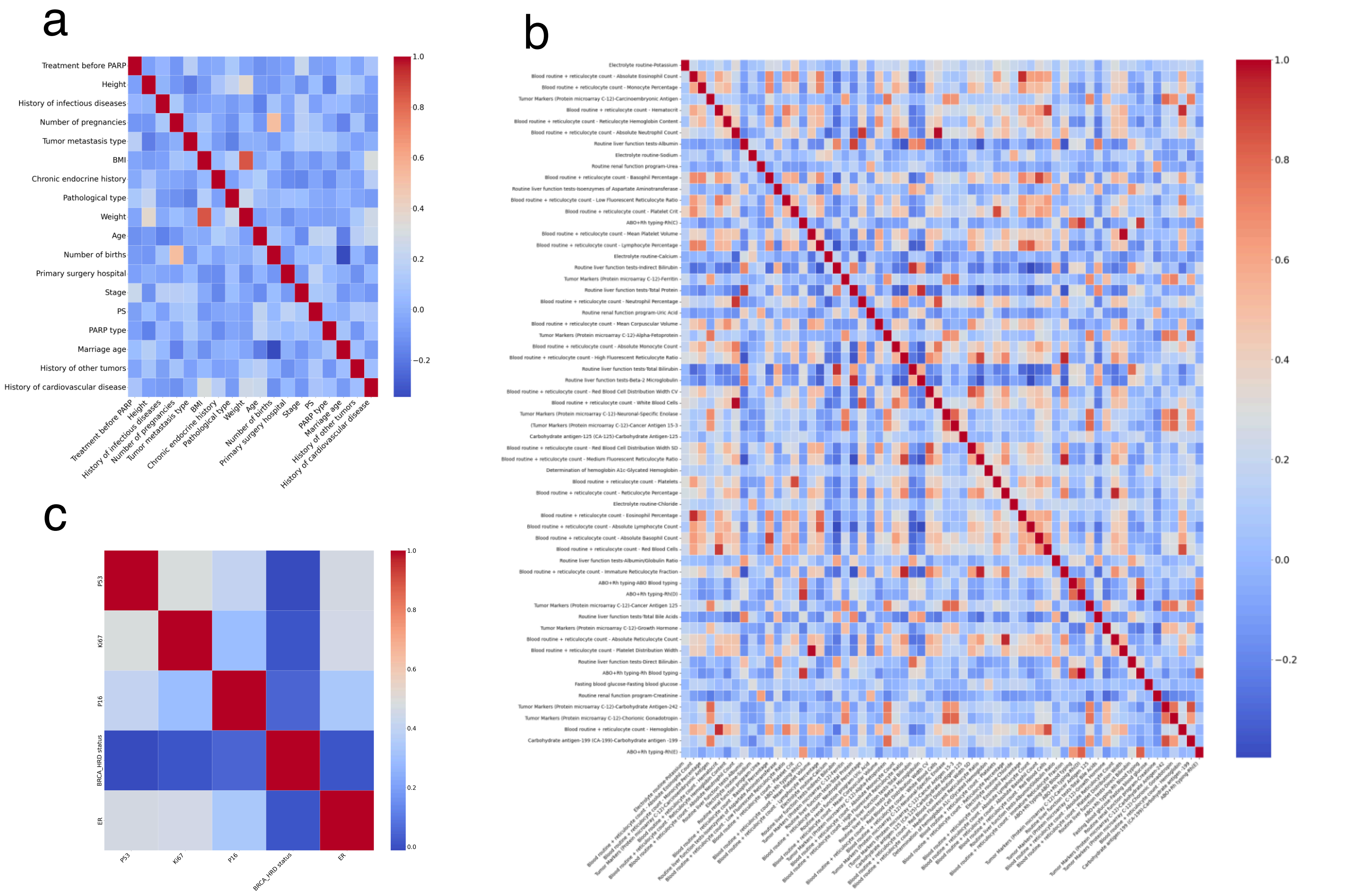
a
Treatment before PARP
Height
History of infectious diseases
Number of pregnancies
Tumor metastasis type
BMI
Chronic endocrine history
Pathological type
Weight
Age
Number of births
Primary surgery hospital
Stage
PS
PARP type
Marriage age
History of other tumors
History of cardiovascular disease
1.0
0.8
0.6
0.4
0.2
0.0
−0.2
b
Electrolyte routine-Potassium
Blood routine + reticulocyte count - Absolute Eosinophil Count
Blood routine + reticulocyte count - Monocyte Percentage
Tumor Markers (Protein microarray C-12)-Carcinoembryonic Antigen
Blood routine + reticulocyte count - Hematocrit
Blood routine + reticulocyte count - Reticulocyte Hemoglobin Content
Blood routine + reticulocyte count - Absolute Neutrophil Count
Routine liver function tests-Albumin
Electrolyte routine-Sodium
Routine renal function program-Urea
Blood routine + reticulocyte count - Basophil Percentage
Routine liver function tests-Isoenzymes of Aspartate Aminotransferase
Blood routine + reticulocyte count - Low Fluorescent Reticulocyte Ratio
Blood routine + reticulocyte count - Platelet Crit
ABO+Rh typing-Rh(C)
Blood routine + reticulocyte count - Mean Platelet Volume
Blood routine + reticulocyte count - Lymphocyte Percentage
Electrolyte routine-Calcium
Routine liver function tests-Indirect Bilirubin
Tumor Markers (Protein microarray C-12)-Ferritin
Routine liver function tests-Total Protein
Blood routine + reticulocyte count - Neutrophil Percentage
Routine renal function program-Uric Acid
Blood routine + reticulocyte count - Mean Corpuscular Volume
Tumor Markers (Protein microarray C-12)-Alpha-Fetoprotein
Blood routine + reticulocyte count - Absolute Monocyte Count
Blood routine + reticulocyte count - High Fluorescent Reticulocyte Ratio
Routine liver function tests-Total Bilirubin
Routine liver function tests-Beta-2 Microglobulin
Blood routine + reticulocyte count - Red Blood Cell Distribution Width CV
Blood routine + reticulocyte count - White Blood Cells
Tumor Markers (Protein microarray C-12)-Neuronal-Specific Enolase
(Tumor Markers (Protein microarray C-12)-Cancer Antigen 15-3
Carbohydrate antigen-125 (CA-125)-Carbohydrate Antigen-125
Blood routine + reticulocyte count - Red Blood Cell Distribution Width SD
Blood routine + reticulocyte count - Medium Fluorescent Reticulocyte Ratio
Determination of hemoglobin A1c-Glycated Hemoglobin
Blood routine + reticulocyte count - Platelets
Blood routine + reticulocyte count - Reticulocyte Percentage
Electrolyte routine-Chloride
Blood routine + reticulocyte count - Eosinophil Percentage
Blood routine + reticulocyte count - Absolute Lymphocyte Count
Blood routine + reticulocyte count - Absolute Basophil Count
Blood routine + reticulocyte count - Red Blood Cells
Routine liver function tests-Albumin/Globulin Ratio
Blood routine + reticulocyte count - Immature Reticulocyte Fraction
ABO+Rh typing-ABO Blood typing
ABO+Rh typing-Rh(D)
Tumor Markers (Protein microarray C-12)-Cancer Antigen 125
Routine liver function tests-Total Bile Acids
Tumor Markers (Protein microarray C-12)-Growth Hormone
Blood routine + reticulocyte count - Absolute Reticulocyte Count
Blood routine + reticulocyte count - Platelet Distribution Width
Routine liver function tests-Direct Bilirubin
ABO+Rh typing-Rh Blood typing
Fasting blood glucose-Fasting blood glucose
Routine renal function program-Creatinine
Tumor Markers (Protein microarray C-12)-Carbohydrate Antigen-242
Tumor Markers (Protein microarray C-12)-Chorionic Gonadotropin
Blood routine + reticulocyte count - Hemoglobin
Carbohydrate antigen-199 (CA-199)-Carbohydrate antigen -199
ABO+Rh typing-Rh(E)
1.0
0.8
0.6
0.4
0.2
0.0
−0.2
c
P53
Ki67
P16
BRCA_HRD status
ER
1.0
0.8
0.6
0.4
0.2
0.0
